# Cytotoxicity, Biocompatibility, and Calcium Deposition Capacity of 45S5 Bioglass Experimental Paste and Bio-C Temp: In Vitro and In Vivo Study Using Wistar Rats

**DOI:** 10.3390/jfb15070184

**Published:** 2024-07-04

**Authors:** Francine Benetti, Pedro Henrique Chaves de Oliveira, Maria Paula Bernal de Andrade, Cristiane Cantiga-Silva, Gustavo Sivieri-Araújo, Eloi Dezan Júnior, João Eduardo Gomes-Filho, Ivana Márcia Alvez Diniz, Alexandre Henrique dos Reis-Prado, Marina Trevelin Souza, Edgar Dutra Zanotto, Luciano Tavares Angelo Cintra

**Affiliations:** 1 Endodontic Section, Department of Restorative Dentistry, School of Dentistry, Universidade Federal de Minas Gerais, Belo Horizonte CEP 31270-901, MG, Brazil; francine-benetti@ufmg.br (F.B.); ivanadiniz@ymail.com (I.M.A.D.); alexandreprado.cba@gmail.com (A.H.d.R.-P.); 2 Endodontic Section, Department of Preventive and Restorative Dentistry, School of Dentistry, São Paulo State University (UNESP), José Bonifácio 1193, Vila Mendonça, Araçatuba CEP 16015-050, SP, Brazil; pedro.chaves@unesp.br (P.H.C.d.O.); mariapaulab@outlook.com (M.P.B.d.A.); cristiane.cantiga@unesp.br (C.C.-S.); gustavo.sivieri@unesp.br (G.S.-A.); eloi.dezan@unesp.br (E.D.J.); joao.eduardo@unesp.br (J.E.G.-F.); 3 Vitreous Materials Laboratory (LaMaV), Department of Materials Engineering, Federal University of São Carlos (UFSCar), Sao Carlos CEP 13565-905, SP, Brazil; marina.trevelin@gmail.com (M.T.S.); dedz@ufscar.br (E.D.Z.)

**Keywords:** cytotoxicity, biocompatibility, biomineralization, intracanal medication, bioglass

## Abstract

The evolution of biomaterials engineering allowed for the development of products that improve outcomes in the medical–dental field. Bioglasses have demonstrated the ability to either compose or replace different materials in dentistry. This study evaluated the cytotoxicity, biocompatibility, calcium deposition, and collagen maturation of 45S5 bioglass experimental paste and Bio-C Temp, compared to calcium hydroxide (Ca(OH)_2_) paste. The 45S5 bioglass and Ca(OH)_2_ powder were mixed with distilled water (ratio 2:1); Bio-C Temp is ready-for-use. Dental pulp cells were exposed to the materials’ extracts (1:2 and 1:4 dilutions; 24, 48, and 72 h) for MTT and live/dead analyses. Polyethylene tubes filled with the pastes, or left empty (control), were implanted on the dorsum of 16 rats. After 7 and 30 days (n = 8/period), the rats were euthanized and the specimens were processed for hematoxylin–eosin (H&E), von Kossa (vK), and picrosirius red (PSR) staining, or without staining for polarized light (PL) birefringence analysis. A statistical analysis was applied (*p* < 0.05). There was no difference in cell viability among Ca(OH)_2_, 45S5 bioglass, and the control, across all periods and dilutions (*p* > 0.05), while Bio-C Temp was cytotoxic in all periods and dilutions compared to the control (*p* < 0.05). Regarding biocompatibility, there was a reduction in inflammation from 7 to 30 days for all groups, without significant differences among the groups for any period (*p* > 0.05). The fibrous capsules were thick for all groups at 7 days and thin at 30 days. All materials showed positive structures for vK and PL analysis. At 7 days, the control and 45S5 bioglass showed more immature collagen than the other groups (*p* < 0.05); at 30 days, 45S5 bioglass had more immature than mature collagen, different from the other groups (*p* < 0.05). In conclusion, Bio-C Temp presented cytotoxicity compared to the other materials, but the three pastes showed biocompatibility and induced calcium deposition. Additionally, the bioglass paste allowed for marked and continuous collagen proliferation. This study contributed to the development of new biomaterials and highlighted different methodologies for understanding the characteristics of medical–dental materials.

## 1. Introduction

It is known that endodontic therapies aim to eliminate bacterial content as well as repair periapical tissues [1]. Calcium hydroxide (Ca(OH)_2_) has been used for a long time due to its ability to dissociate into calcium and hydroxyl ions, which increases pH, favoring its biological and microbiological properties [2]. Additionally, with calcium dissociation, it is possible to observe the induction of mineralized tissue [3]. Moreover, its bioactivity is confirmed by allowing calcium deposition and tissues cell proliferation [4,5,6]. However, Ca(OH)_2_-based pastes have low radiopacity and flow capacity, which makes them difficult to insert into the root canal [7], and when used for prolonged periods, e.g., for 6 months, they can induce tissue degradation by disrupting the collagen fibers, which can weaken the dentin and increase the risk of tooth fracture [8]. Thus, endodontic science seeks new materials to overcome these disadvantages.

In this regard, studies have been evaluating bioglasses as viable alternatives. These materials possess great potential for tissue regeneration, as they can induce the formation of collagenous and bone tissues, and even new blood vessels [9]. The first bioglass composition is known as 45S5 bioglass, developed by Hench in 1969 [10], and it consists of 46.1% SiO_2_, 24.4% Na_2_O, 26.9% CaO, and 2.6% P_2_O_5_ (mol%). In addition to having excellent bioactive properties [9], it has reasonable mechanical strength and the ability to withstand stress and does not suffer from corrosion [11]. 45S5 bioglass has widely demonstrated its potential for tissue regeneration and stimulating cell differentiation [12]. The biological activity is associated with the processes of apatite hydroxycarbonate production, which forms in contact with an aqueous medium and is related to the production of mineralized tissue [13]. These facts provide outstanding potential for this biomaterial to be used in the dental field [14]. As an intracanal medication in endodontics, a previous study evaluated other formulations of bioglass, and the results were promising [4]. Therefore, it is of great interest to evaluate this bioglass in a paste form to be used in infected root canals.

Another biomaterial that has been studied is Bio-C Temp (Angelus, Londrina, PR, Brazil), and few studies have explored its biological properties [15,16,17,18,19]. Bio-C Temp is a bioceramic material indicated as intracanal medication in cases of conventional endodontic treatment, apexification, and endodontic regeneration [18]. This material demonstrated the capacity to release calcium ions [17], antimicrobial properties [18], and acceptable radiopacity [15]. Although few studies have evaluated the cytotoxicity of this material, their design and methodologies did not allow a complete in vitro and in vivo evaluation of the material. Villa et al. [15] did not compare its cytotoxicity with other materials; Oliveira et al. [16] evaluated only one experimental period, which may limit the result analysis; Guerreiro et al. [18] evaluated it on osteoblast-like cells; and Oliveira et al. [17] compared Bio-C Temp with reparative sealers and not with intracanal medications. A single study evaluated its biocompatibility in the subcutaneous tissue of rats, verifying its inflammatory process and the maturation of collagen fibers [19]. However, the calcium deposition capacity has not been evaluated yet, which is a very relevant property for an intracanal medication since this may indicate the ability of this material to induce tissue biomineralization [4].

Therefore, considering the lack of studies and specific outcomes in vitro and in vivo of the 45S5 bioglass in the form of a paste for intracanal medication, and the need for further studies on the Bio-C Temp paste, this study aimed to evaluate the cytotoxicity, biocompatibility, collagen maturation, and calcium deposition of these materials compared to Ca(OH)_2_ paste. The null hypothesis is that there are no differences among these intracanal medication pastes.

## 2. Materials and Methods

### 2.1. Cytotoxicity Analysis

#### 2.1.1. Preparation of Paste Extracts

The powders of 45S5 bioglass (Laboratory of Vitreous Materials, at the Federal University of São Carlos, SP, Brazil) and Ca(OH)_2_ (Biodinâmica Química e Farmacêutica Ltd.a., Ibiporã, PR, Brazil) were obtained, and the pastes were prepared by spatulating each powder with distilled water in a 2:1 ratio by weight [4]. The Bio-C Temp was obtained ready-for-use (Table 1).

Paste extracts were prepared following previous investigations [15] and according to ISO 10993-5 (2009) [20]: 100 mg of Bio-C Temp was added to 1 mL of Dulbecco’s Modified Eagle’s medium (DMEM) with 10% fetal bovine serum. The 1:2 and 1:4 dilutions were used in this study [21].

#### 2.1.2. Dental Pulp Cell Culture

Primary dental pulp cell (DPC) cultures were obtained from Wistar rats in an earlier study authorized by the local ethical committee (CEUA 0094/21). The DPCs were cultured under standard cell culture conditions in DMEM [22] supplemented with 10% fetal bovine serum (FBS), penicillin, and streptomycin, at 37 °C, 100% humidity, 95% air, and 5% CO_2_. The cells were subsequently seeded in 96-well plates (10^4^ cells/well) and incubated for 24 h under standard cell culture conditions to allow the cells to attach to the bottom of each well before adding the solutions. Afterwards, dilutions of the extracts of each material were applied to the cells. Cell viability was assessed using the 3-(4,5-dimethylthiazol-2-yl)-2,5-diphenyltetrazolium bromide (MTT) assay [23] after 24, 48, and 72 h. For this, the culture medium and dilution of the extracts from each well were removed, and 100 µL of MTT solution (0.5 mg/mL) in DMEM without FBS (1:10) was added to each well. The MTT solution was removed after 4 h of incubation, and the formazan crystals were dissolved in 100 μL of isopropyl alcohol. The plate was left at room temperature in a dark room for 30 min on a rotary shaker. The absorbance of the plates was assessed at 570 nm using an Elisa reader (Eon Microplate Spectrophotometer, Biotek, Miami, FL, USA). Each condition was analyzed in triplicate.

In addition, representative epifluorescence images from the live/dead assay were obtained according to the manufacturer’s instructions, to show cell viability at 72 h of the experiment. The analysis was performed in a Cytation multimode reader (Biotek) at 488 nm (live—calcein) and 535 nm (dead—propidium iodide).

### 2.2. In Vivo Study

#### 2.2.1. Subcutaneous Implants

Sixteen 2-month-old male rats (*Rattus albinus*, Wistar), weighing 250–280 g, were used in this study and were kept in an environment with temperature (22–24 °C) and light control (12 h light–dark cycle), receiving water and food ad libitum. The study was approved by the local ethical committee (CEUA 0024/19). The number of animals was established following the protocols of previous studies, using an alpha error of 0.05% and 95% power to recognize a significant difference of 1 in the median scores, with a minimum of seven animals per group considered necessary. Considering that complications could occur, resulting in the death of animals, one more rat was added for each group/period.

After anesthesia, the animals had their dorsa shaved, and following antisepsis with a 5% iodine solution, a 2 cm incision was made in a head-to-tail orientation with a #15 blade. Spaces in the subcutaneous tissue were then created on each side of the incision for the implantation of three tubes filled with the materials and one empty for control. Sixty-four polyethylene tubes (Abbot Lab. Do Brasil Ltd.a., São Paulo, SP, Brazil) with 1.0 mm internal diameters, 1.6 mm external diameters, and 10.0 mm lengths [20] were filled with the materials or left empty for the control [24]. The tubes were immediately implanted into the connective tissue, and the skin was sutured with 4–0 silk sutures. After the implantation, the animals received a dose of 150 mg/Kg of sodium dipyrone and were monitored until they were woken up and began feeding themselves.

After 7 and 30 days (n = 8/period), the animals were euthanized by anesthetic overdose, had the tubes and the surrounding tissue removed, and were fixed in 10% buffered formalin at pH 7.0. The samples were processed; embedded in paraffin; and serially sectioned at 5 μm for hematoxylin and eosin staining and 10 μm for staining according to the von Kossa’s technique, or left unstained to be observed under polarized light.

#### 2.2.2. Tissue Response

A histological analysis was performed by a single calibrated operator in a blinded manner using light microscopy (DM 4000 B; Leica Microsystem, Wetzlar, Germany). The tissue inflammation was graded as follows: “0”, none or minimal inflammatory cells and no reaction; “1”, fewer than 25 cells and a mild reaction; “2”, between 25 and 125 cells and a moderate reaction; and “3”, 125 or more cells and a severe reaction [5]. Fibrous capsules were considered thin when the thickness was less than 150 μm and thick when equal to or greater than 150 μm [4]. Positive structures for von Kossa and polarized light were recorded as absent or present [24].

The maturation levels of the collagen fibers were assessed in sections stained with picrosirius red (PSR) under polarized light microscopy. Greenish-yellow fibers were considered immature and thin; while yellowish-red fibers were classified as mature and thick. After color selection, the software automatically calculated the marked area of each collagen fiber type (Leica QWin V3, Leica Microsystems, Waltham, MA, EUA).

### 2.3. Statistical Analysis

The data were analyzed using SigmaPlot 12.0^TM^ software (SPCC, Inc., Chicago, IL, USA). The Shapiro–Wilk test of normality was applied, the Kruskal–Wallis test followed by Dunn’s test was performed for nonparametric data, and an analysis of variance followed by the Tukey’s test was performed for parametric data. Significance was set at the level of 5% (*p* < 0.05).

## 3. Results

### 3.1. Cell Viability and Live/Dead Assay

**Figure 1:**
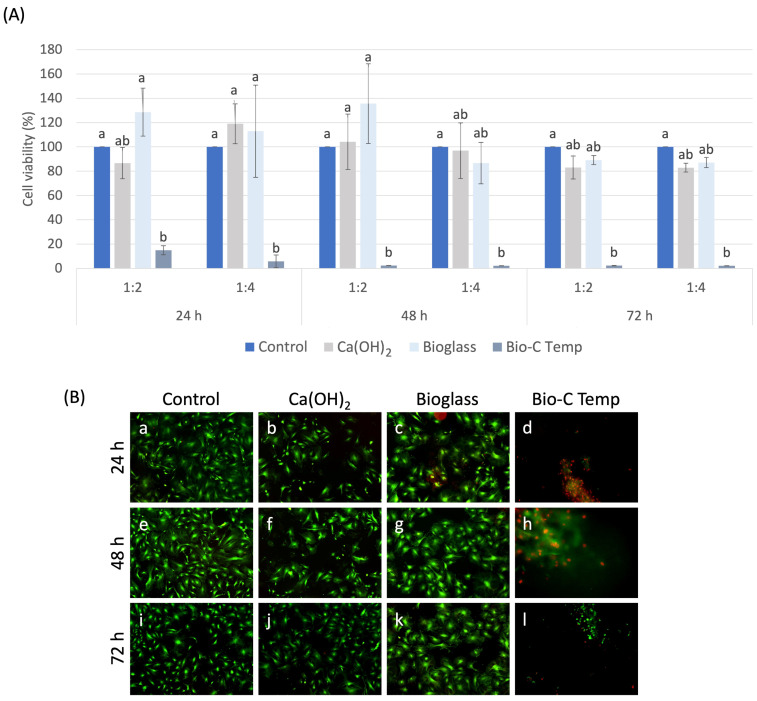
(**A**) Graph representing the viability results of DPCs determined by an MTT assay. The Ca(OH)_2_ and 45S5 bioglass groups showed similar cell viability to the control group, with no significant difference among groups in all analysis periods and extracts evaluated (*p* > 0.05), whereas Bio-C Temp showed high cytotoxicity compared to the other groups (*p* < 0.05). (**B**) Representative epifluorescence images from the live/dead assay, where compromised cells were stained red and live cells were stained green, demonstrating cell viability in the control (**a**,**e**,**i**), Ca(OH)_2_ (**b**,**f**,**j**), and 45S5 bioglass (**c**,**g**,**k**) and Biop-C Temp (**d**,**h**,**l**) at 24 h, 48, and 72 h in the 1:4 dilution.

Cell viability and live/dead assay data are shown in Figure 1. There was no difference in cell viability between the Ca(OH)_2_ and 45S5 bioglass groups, among the groups and the control group, or in any periods or extracts evaluated (*p* > 0.05). Bio-C Temp was more cytotoxic than the control group in all periods of analysis and dilutions (*p* < 0.05). Additionally, it was more cytotoxic than 45S5 bioglass at 24 h in the 1:2 and 1:4 dilutions and at 48 h in the 1:2 dilution (*p* < 0.05). At 72 h, the wells presented dead cells.

### 3.2. In Vivo Analysis

**Figure 2:**
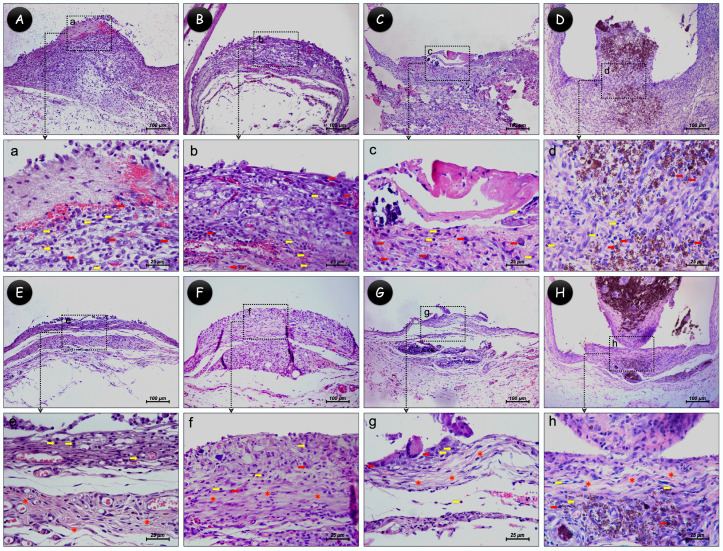
Representative images of inflammatory reaction. (**Aa**–**Dd**) At 7 days: (**Aa**) control, (**Bb**) Ca(OH)_2_, (**Cc**) 45S5 bioglass, and (**Dd**) Bio-C Temp groups with moderate inflammatory cell infiltration and thick fibrous capsule. (**Ee**–**Hh**) At 30 days: (**Ee**) control, (**Ff**) Ca(OH)_2_, (**Gg**) 45S5 bioglass, and (**Hh**) Bio-C Temp groups with mild inflammatory cell infiltration and thin fibrous capsule at the tube opening. Yellow arrows indicate lymphocytes; red arrows indicate macrophages; red asterisks indicate collagen fibers; and black lines show the thickness of the fibrous capsules. ((**A**–**H**) 100×; (**a**–**h**) 400×; hematoxylin–eosin staining).

Representative images of the histological analysis are displayed in Figure 2 and the scores attributed to each group in Table 2. At 7 days, the control group showed a moderate inflammatory infiltrate containing lymphocytes and macrophages, and a thick and disorganized fibrous capsule. All materials also showed moderate inflammatory infiltrate and thick capsules containing mainly macrophages. At 30 days, there was more organized fibrous capsules with few chronic inflammation cells in all groups, representing a discreet inflammatory infiltrate. Furthermore, a greater number of blood vessels and fewer collagen fibers were observed at 7 days for all groups; at 30 days, there was an apparent reduction in vascularization and an increase in collagen fibers in the organized fibrous capsules. There was no significant difference between groups in any period of analysis (*p* > 0.05).

**Figure 3:**
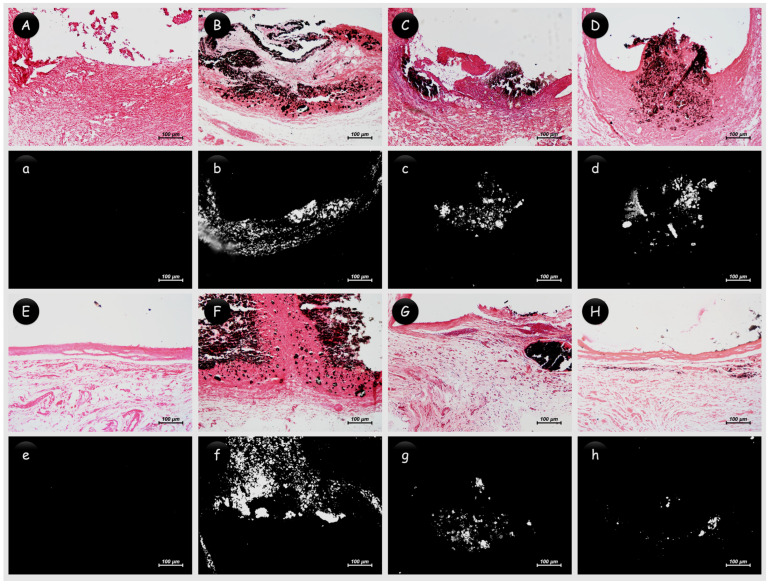
Representative images of biomineralization analysis. Positive structures for von Kossa can be seen in the images as black structures, and positive structures for polarized light are observed as birefringent structures. (**A**–**H**) Images of von Kossa and (**a**–**h**) polarized light analysis. (**Aa**–**Dd**) Day 7 and (**Ee**–**Hh**) day 30. (**Aa**, **Ee**) Control group with absence of positive structures; (**Bb**, **Ff**) Ca(OH)_2_, (**Cc**, **Gg**) 45S5 bioglass, and (**Dd**, **Hh**) Bio-C Temp groups with presence of positive structures for both analyses. ((**A**–**H**) 100×; von Kossa staining. (**a**–**h**) 100×; polarized light visualization).

**Figure 4:**
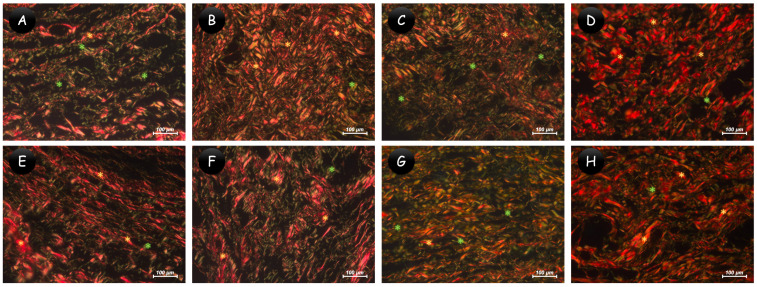
Representative images of collagen maturation. Greenish-yellow fibers are considered immature and thin (green asterisks), while yellowish-red fibers are considered mature and thick (yellow asterisks). (**A**–**D**) At 7 days: (**A**) control and (**C**) 45S5 bioglass groups showing more immature fibers; (**B**) Ca(OH)_2_ and (**D**) Bio-C Temp groups showing more mature fibers. (**E**–**H**) At 30 days: (**E**) control, (**F**) Ca(OH)_2_, and (**H**) Bio-C Temp groups showing more mature fibers; (**G**) 45S5 bioglass showing more immature fibers. ((**A**–**H**) 400×; picrosirius red staining).

Representative images of the von Kossa analysis or polarized light are presented in Figure 3. All the biomaterials tested showed positive structures for von Kossa, visible in the images as black structures, and positive structures for polarized light, observed as birefringent structures (Table 2). The control group was not positive for this analysis. The data for collagen maturation are summarized in Table 3, with representative images shown in Figure 4. At 7 days, the control and 45S5 bioglass groups had more immature collagen fibers compared to the Ca(OH)_2_ and Bio-C Temp groups (*p* < 0.05); additionally, Bio-C Temp had fewer immature fibers compared to all groups (*p* < 0.05). At 30 days, only 45S5 bioglass had more immature than mature fibers, differing from the other groups (*p* < 0.05).

## 4. Discussion

This study evaluated the cytotoxicity, biocompatibility, potential for calcium deposition, and the maturity of collagen fibers induced by a paste produced from 45S5 bioglass and the ready-to-use paste Bio-C Temp. The two pastes were compared to conventional Ca(OH)_2_ paste, and both pastes showed differences in the in vitro and in vivo studies. Thus, the null hypothesis was rejected.

Firstly, in the cytotoxicity analysis, it was observed that the 45S5 bioglass paste presented similar results to the Ca(OH)_2_ paste. However, the Bio-C-temp paste presented more pronounced cytotoxicity in the different extracts and periods of analysis compared to the other groups. This test was performed with primary dental pulp cells extracted from rats at different concentrations and periods, which is an important method to understand the behavior of pulp cells when in contact with these different materials [25].

The cytotoxicity of 45S5 bioglass has already been evaluated in association with different compounds [26,27]. In most studies, it was demonstrated that 45S5 bioglass allows cell growth with low toxicity. Silver et al. [28] demonstrated that 45S5 bioglass had no effect on osteoblast viability, and under most conditions, did not affect either proliferation or differentiation [27]. Bakry et al. [29] evaluated the cytotoxicity of a paste composed of 45S5 bioglass mixed with 50% phosphoric acid (H_3_PO_4_) that was indicated for dentin hypersensitivity treatment. It was found that 45S5 cytotoxicity was higher than that for the control, differing from the results of the present study. However, the presence of H_3_PO_4_ must be considered, which may have increased the toxicity of the paste. In paste form, 45S5 bioglass has been investigated essentially for enamel remineralization [30,31], which prevents a direct comparison with our results.

Other biocomposites have been studied for the field of dentistry [32,33]. For example, a synthetic and bioactive osteoplastic powder was evaluated for the recovery of bone defects in oral and maxillofacial surgery, presenting the ability to integrate into the area of the alveolar bone defect without presenting systemic toxicity in rats. This biocomposite was based on an original sol–gel synthesis [33]. The use of the sol–gel method has been widely employed to produce bioglasses due to the high surface area and porosity that can be obtained [32,34]. It is therefore possible to produce a bioglass with greater bioactive capacity [34]. The sol–gel method involves the production of bioglass at low temperatures, unlike the conventional method, which involves high temperatures, as occurs with the 45S5 bioglass used in our study. However, our study showed that conventionally produced bioglass has the potential to be used in dentistry as a paste from its powder, as it allows cell viability, induction of biomineralization, and collagen proliferation.

Some previous studies evaluated the cytotoxicity of Bio-C Temp, nevertheless using different experimental designs and methodologies. Villa et al. [15] performed the first cytotoxicity assay with Bio-C Temp but did not compare it with other materials. On the other hand, it was shown that Bio-C Temp exhibited dose- and time-dependent cytotoxicity, in addition to the absence of penetration into dentinal tubules [15]. Oliveira et al. [16] demonstrated that at dilutions of 1:1 and 1:2, Bio-C Temp had significantly lower viability compared to other dilutions and the control group, as well as when compared to MTA Flow and UltraCal XS. Although only 24 h was used as the experimental time, this study showed the cytotoxic potential of the Bio-C Temp paste [16]. Similarly, in the 24-h period of the present study, Bio-C Temp was more cytotoxic compared to the control and 45S5 bioglass paste at 1:2 dilution and more cytotoxic than all groups at 1:4 dilution. Furthermore, in the present study, the Bio-C Temp paste also showed greater cytotoxicity compared to the control at 48 and 72 h. This new finding demonstrates that this paste continues to release compounds that may interfere with cell viability over longer periods of observation.

In a study employing osteoblast-like cells, Guerreiro et al. [18] did not observe any difference in the cell’s viability exposed to different intracanal drug extracts, except in the 1:2 dilution, in which the Bio-C Temp group showed significantly lower cell viability than UltraCal XS in the MTT assay [18]. Oliveira et al. [17] compared Bio-C Temp with reparative sealers, differing from this study, which compared it with intracanal medications. However, as shown in the present study, Bio-C Temp had a lower percentage of cell viability than the control at all dilutions tested.

This study also evaluated the biocompatibility in the subcutaneous tissue of rats, and it was possible to observe that the 45S5 bioglass, Bio-C Temp, and Ca(OH)_2_ pastes induced a similar inflammatory infiltrate at 7 and 30 days. The outcomes for the different pastes were similar to these obtained in the control group at 30 days, showing that they are biocompatible. When the materials are in close contact with the tissue, they can induce some degree of inflammation; however, the stimulus dwell time is more important than the irritating potential of the material [35,36]. The difference between the results of the cytotoxicity assay and biocompatibility studies allows us to understand that even if Bio-C Temp was shown to be cytotoxic, the tissues can tolerate it well over time.

In this study, Ca(OH)_2_ paste was used to compare the reaction of the other pastes, as it is widely used in endodontics. Initially, this material causes moderate inflammation due to its potential to promote necrosis in tissues at first contact; however, a significant reduction in the number of inflammatory cells and giant cells was observed after 30 days [7]. For glass–ceramics or bioglass, a single previous study, carried out by de Araújo Lopes et al. [4], studied two-phased Biosilicate and F18 bioactive glass as pastes for intracanal medication. It was found that two-phased Biosilicate and Ca(OH)_2_ pastes induced moderate inflammation seven days after implantation, while F18 bioactive glass was associated with moderate to severe inflammation; at 30 days, most specimens from the control, F18, and two-phased Biosilicate groups showed mild inflammation, while Ca(OH)_2_ showed mild to moderate inflammation [4]. These results are associated with tissues’ initial interactions with the materials, which initially provoked pronounced inflammation but started to allow ionic exchanges over time, which led to the formation of bioactive compounds and the proliferation of specific cells that the material is associated with, decreasing the inflammation and inducing repair [10,37]. These results are similar to those found in this study, indicating the potential for different bioglass formulations to be well tolerated by tissues [37] and as an alternative material to be used as intracanal medication [4].

Another relevant analysis performed herein is the quantification of mature collagen fibers. It was observed that at 7 days, the 45S5 bioglass had a higher number of immature fibers, as did the control. This result may be related to its lower-intensity inflammatory response, allowing the proliferation of younger fibers quickly. At 30 days, the 45S5 bioglass still had a higher number of immature fibers, which may be related to constant tissue proliferation, a fact that can be verified in other studies [12,28,38]. Thus, we can correlate collagen maturation, which in part indicates the reparative process, with the ability of 45S5 bioglass to allow and stimulate tissue growth [11]. For Bio-C Temp, Lopes et al. [19] demonstrated that the maturation of collagen fibers is associated with the inflammatory profile. The present study linked collagen maturation with cell viability, biocompatibility, and calcium deposition, exploring through a picrosirius red analysis, which indicates immature or mature collagen fibers related to tissue repair process [39].

Regarding bioactivity, studies have shown that bioceramic materials in odontology possess the capability to enhance osteoblastic differentiation and promote mineralization in cell lines [3,4,18]. Bio-C Temp demonstrated higher alkaline phosphatase activity and calcium nodule deposition [18]. Ca(OH)_2_ pastes are known to create an alkaline pH environment necessary for initiating biological processes. This alkaline pH facilitates the activation of alkaline phosphatase, enabling phosphate to react with released calcium ions from Ca(OH)_2_’s ionic dissociation, forming calcium phosphate, which is crucial for mineralization processes [3,18]. The 45S5 bioglass can promote ionic binding when in contact with surrounding tissues, enabling the formation of hydroxyapatite, a compound associated with bone preformation [11]. Rizwan et al. [38] studied the ability of this material to bind bones and soft tissues. The authors confirmed its biocompatibility and tissue formation capacity [37]. This information is consistent with the findings of this research, showing low cytotoxicity, good biocompatibility, and low maturation of collagen, in addition to the ability to deposit calcium, as observed by the von Kossa technique.

The ability of Bio-C Temp in terms of calcium deposition was demonstrated in a previous study, conducted in vitro [15,16]. In the present study, positive depositions in the von Kossa technique and birefringent granulations under polarized light were observed, indicating the biomineralization capacity of this material. Bio-C Temp contains tricalcium silicate, dicalcium, and calcium oxide, which when hydrated in the oral environment, can form Ca(OH)_2_ that quickly releases calcium and hydroxyl ions [15]. As is known, the dissociation of these ions increases the pH levels of the medium, acting as an antibacterial and calcium favoring bone formation [15].

The presence of calcium deposition verified by the von Kossa technique confirms the presence of calcium carbonate in the tissue, which is presented as calcite, originating from the reaction of the material’s calcium and the tissue’s carbon dioxide [24,36]. Adjacent to the calcite granulations, fibronectin protein begins to accumulate, leading to the formation of dystrophic calcifications [40,41]. The polarized light technique allows for the observation of birefringent calcium crystals, which also originated from the combination of ions released from the material and tissues, showing calcium crystals that allow us to identify a hydroxyapatite formation process [24,40]. The presence of calcium carbonate was observed in the Ca(OH)_2_, 45S5 bioglass, and Bio-C Temp groups based on the von Kossa and birefringent granulations under polarized light at 7 and 30 days, findings not observed in the control group. Thus, it is stated that all materials showed the ability to induce calcium deposition in vivo in all periods of analysis.

## 5. Conclusions

This study shows different results explored through in vitro and in vivo methodologies. The cytotoxicity, biocompatibility, calcium deposition potential, and maturity of collagen fibers induced by medications used between sessions during the endodontic treatment of infected teeth were evaluated. An experimental paste produced from 45S5 bioglass, the ready-to-use Bio-C Temp paste, and the conventional Ca(OH)_2_-based paste were comparatively evaluated. In the cytotoxicity test carried out on primary dental pulp cells extracted from rats, it was observed that the 45S5 bioglass paste presented similar results to the Ca(OH)_2_ paste, while Bio-C-temp presented more pronounced cytotoxicity. On the other hand, all three pastes showed biocompatibility and the ability to induce calcium deposition. Notably, the experimental 45S5 bioglass paste facilitated a marked and continuous proliferation of collagen fibers, indicating its significant beneficial tissue response. Furthermore, unlike previously published findings, the materials showed biological interaction with cells and tissues across all periods of analysis, demonstrating tissue tolerance and repair capacity. These findings contribute to science and the development of new biomaterial alternatives based on bioglass, as well as expand on the knowledge and perspectives for researchers and clinicians, particularly in the field of endodontics.

## Figures and Tables

**Table 1:** Intracanal medications, manufacturers, compositions, and technical information.

Groups	Manufacturer	Composition	Technical Information
Ca(OH)_2_	Sigma-Aldrich, San Luis, Missouri, EUA	Ca(OH)_2_	Prepared by spatulation with distilled water in a 2:1 ratio of by weight
45S5 bioglass	Laboratório de Materiais Vítreos (LaMaV), São Carlos, Brazil	46.1% SiO_2_, 24.4% Na_2_O, 26.9% CaO, and 2.6% P_4_O_5_ (mol%)	Prepared by spatulation with distilled water in a 2:1 ratio of by weight
Bio-C Temp	Angelus Indústria de Produtos Odontológicos S/A, Londrina, PR, Brazil	Ca_2_O_4_Si, CaAl_2_O_4_, CaO, CaWO_4_, TiO_2_ and dispersing agent	Paste ready for use

**Table 2:** Scores and median for inflammatory infiltrate, fibrous capsule thickness, and biomineralization for all groups and periods of analysis.

Time	Groups *	Scores				Median	Fibrous Capsule	vK/PL	*p* Value
		1	2	3	4				
7 days	Control ^a^	0	3	4	1	3	Thick	0	0.361
	Ca(OH)_2_^a^	0	1	4	3	3	Thick	100	
	45S5 bioglass ^a^	0	3	4	1	3	Thick	100	
	Bio-C Temp ^a^	0	1	5	2	3	Thick	100	
30 days	Control ^a^	2	4	2	0	2	Thin	0	0.283
	Ca(OH)_2_^a^	0	5	3	0	2	Thin	100	
	45S5 bioglass ^a^	2	5	1	0	2	Thin	100	
	Bio-C Temp ^a^	3	4	1	0	2	Thin	100	

* One-way analysis of variance; same superscript letters indicate an absence of statistical difference among the groups in each analysis period (*p* > 0.05).

**Table 3:** Percentages of immature and mature collagen fibers in the fibrous capsule according to group at 7 and 30 days.

Groups	Collagen Fibers (%)			
	7 Days *		30 Days ^≠^	
	Immature	Mature	Immature	Mature
Control ^Aa^	65.00 ± 10.03	35.00 ± 10.03	34.94 ± 19.32	65.06 ± 19.32
Ca(OH)_2_^Ba^	27.30 ± 8.63	72.70 ± 8.63	31.89 ± 6.80	68.11 ± 6.80
45S5 bioglass ^Ab^	74.71 ± 8.42	25.29 ± 8.42	86.30 ± 1.27	13.70 ± 1.27
Bio-C Temp ^Ca^	12.71 ± 6.12	87.29 ± 6.12	15.33 ± 7.47	84.67 ± 7.47
*p* value	<0.001		<0.001	

One-way ANOVA test followed by Tukey’s test, after a normality test; Same uppercase (*) and lowercase (^≠^) letters indicate no statistical difference among the groups at 7 and 30 days, respectively (*p* > 0.05).

## Data Availability

No new data were created or analyzed in this study. Data sharing is not applicable to this article.
